# Haematologic and Urologic Manifestations of *Cryptococcus neoformans*

**DOI:** 10.1155/2024/2245391

**Published:** 2024-05-10

**Authors:** Jessica Morgan, John Vardanega, Mimi Yue, Ian Gassiep

**Affiliations:** ^1^Infectious Diseases, Department of Medicine, Mater Public Hospital, South Brisbane, Queensland, Australia; ^2^Haematology, Department of Medicine, Mater Public Hospital, South Brisbane, Queensland, Australia; ^3^Faculty of Medicine, University of Queensland, Brisbane, Australia; ^4^Pathology Queensland, Royal Brisbane & Women's Hospital, Herston, Queensland, Australia

## Abstract

*Cryptococcus neoformans* classically causes pulmonary and central nervous system (CNS) infection in immunocompromised hosts and can lead to disseminated disease. Two cases of atypical cryptococcal infection are presented—an elderly Human Immunodeficiency Virus- (HIV-) negative male with a urinary source of infection and a young HIV-positive male with bone marrow infiltration complicated by haemophagocytic lymphohistiocytosis (HLH). A literature review of systemic cryptococcal infections involving the genitourinary tract and bone marrow was performed. These cases highlight the importance of clinicians considering uncommon manifestations of cryptococcal disease.

## 1. Background

Invasive fungal infections have varied manifestations and can cause significant morbidity and mortality, making their timely diagnosis crucial. Cryptococcal infection classically occurs following inhalation of environmental spores leading to presentations with predominantly pulmonary and CNS infections [1]. *Cryptococcus neoformans* is an important cause of invasive fungal infection in immunocompromised hosts [1]. Whilst the association between cryptococcosis and HIV infection is well established, there is an increasing recognition of underlying systemic disorders causing susceptibility to cryptococcal infection, such as immune deficiencies, organ failure, and various haematological conditions [2–4]. The first case presented highlights underlying haematological disease as one such risk factor. The second case adds to the increasingly recognised link between cryptococcal infection and HLH in the setting of HIV infection [5]. Among the various clinical manifestations of cryptococcosis, primary urinary infection and bone marrow infiltration are rare. Both cases presented highlight examples of these two unusual clinical presentations.

## 2. Case Presentation

### 2.1. Case 1

An older male from regional Queensland, Australia, presented to a tertiary centre via interhospital transfer. His predominant symptoms were that of fever and abdominal pain. His computed tomography (CT) imaging identified an infected obstructed right kidney complicated by a mid-ureteric calculus requiring urgent ureteric stenting. The patient was febrile postoperatively but on examination had no focal neurological deficits, respiratory symptoms, or ocular disease. Significant medical history included chronic kidney disease (CKD) and prefibrotic myelofibrosis (JAK2 V617F positive) which progressed to fibrotic myelofibrosis, managed with pegylated interferon alfa-2a. He had no significant environmental or animal exposures.

### 2.2. Investigations

Urine culture collected at a regional hospital prior to transfer was initially reported as having a moderate growth of *Candida* species based on colonial morphology, without a formal identification or susceptibility testing performed. Three sets of peripheral blood cultures collected pre- and postoperatively as part of septic screens flagged positive with Gram stains resembling yeast cells, and culture subsequently identified the organism as *Cryptococcus neoformans.* Following the blood culture identification, the urine isolate was revisited and subsequently identified as *C. neoformans* by VITEK® mass spectrometry (bioMérieux, Marcy-l'Etoile, France) and Canavanine glycine bromothymol blue (CGB) agar. A serum cryptococcal antigen titre (IMMY Inc., Norman, OK) was performed on serum at admission and found to be positive at 1 : 16. HIV serology was negative. A bone marrow aspirate and trephine (BMAT) was performed which did not demonstrate growth on fungal cultures. Cerebrospinal fluid (CSF) testing did not detect *C. neoformans* via nucleic acid amplification testing (AusDiagnostics Multiplex CSF panel (Mascot, Australia)), was cryptococcal antigen negative, and yielded no growth on fungal cultures. Further CT imaging did not demonstrate any lesions suspicious for intracranial, intrathoracic, or intraabdominal cryptococcal disease, supporting the unusual assessment that the clinical presentation did not involve pulmonary or CNS disease.

### 2.3. Management and Outcome

The patient was initially commenced on intravenous fluconazole and caspofungin as empiric therapy for presumed candidaemia given the clinical presentation with urosepsis and initial urine culture report. Two days later, this was escalated to a six-week course of intravenous liposomal amphotericin B (L-AMB) and oral flucytosine (5-FC) for *Cryptococcus neoformans* cover. He was then transitioned to a consolidation phase of fluconazole 800 mg daily given the organism's elevated minimum inhibitory concentration (MIC) of 8 to fluconazole. Although this dose was planned for 6–8 weeks, it required early de-escalation due to a severe drug-induced liver injury.

The patient was eventually discharged home and continued on oral fluconazole, with a plan to complete a minimum of 12 months of therapy, before commencing secondary prophylaxis given his ongoing immunosuppression.

### 2.4. Case 2

A young adult male presented to a regional Queensland hospital with headache, dyspnoea, and malaise. He developed haemodynamic instability, leading to interhospital transfer to a metropolitan tertiary centre and intensive care unit (ICU) admission. His initial neurological examination was initially unremarkable, but he later developed bilateral lower limb hyperreflexia and ankle clonus. He had no significant past medical history.

### 2.5. Investigations

**Figure 1:**
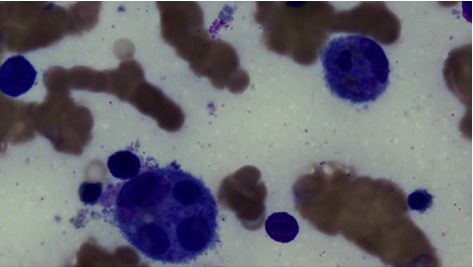
Bone marrow aspirate showing haemophagocytosis, Wright-Giemsa stain, ×100 (courtesy of Dr. Mimi Yue).

**Figure 2:**
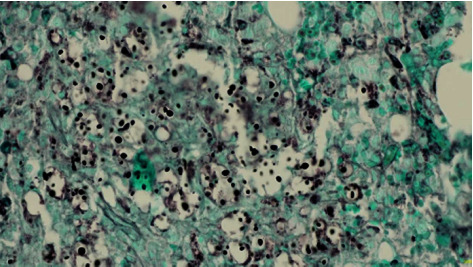
Bone marrow trephine biopsy showing cryptococcal infiltrate. Paraffin-embedded, Grocott methenamine silver stain, ×40 (courtesy of Dr. Cyriac Abraham).

CT imaging demonstrated diffuse interstitial pulmonary infiltrates, bilateral pleural effusions, and right-sided patchy ground glass change. Moderate volume ascites and splenomegaly were also identified. HIV serology was positive, with an initial HIV viral load of 4.29 million copies/millilitre and a CD4 lymphocyte count of 10. A cytomegalovirus (CMV) viraemia with 769 copies/millilitre was identified without a clear focus of end-organ disease. *Pneumocystis jirovecii* (PJP) nucleic acids were not detected on a single salivary sputum specimen and testing for SARS-CoV-2 was negative. Peripheral blood cultures at admission flagged positive with Gram staining identifying budding yeast cells. *C. neoformans* was subsequently identified, which continued to grow from blood cultures for a further 8 days despite antifungal therapy with cryptococcal activity. Serum and CSF cryptococcal antigen titres were both >1 : 1024. Lumbar puncture revealed an opening pressure exceeding 35 cm H_2_O, a positive India ink stain, and *C. neoformans* isolated from culture. Multiple subsequent therapeutic lumbar punctures were performed for persistent headache secondary to raised intracranial pressure (ICP). The patient's serum ferritin level was incidentally noted to be elevated through haematinics for investigation of his significant anaemia. His ferritin peaked at 13,300 *μ*g/L with a diagnosis of HLH confirmed using H score criteria, through which his score was 276 points with a 99% probability of HLH [6]. A subsequent BMAT identified haemophagocytosis, numerous budding yeast on Grocott methenamine silver stain, and *C. neoformans* was again cultured (Figures 1 and 2). Mycobacterial cultures failed to grow acid-fast organisms.

A summary of the investigations of both cases is presented in Table 1.

### 2.6. Management and Outcome

The patient initially received broad-spectrum empiric antimicrobials for pneumonia and sepsis. Upon isolation of *C. neoformans*, intravenous L-AMB and oral 5-FC were commenced. The patient was treated for disseminated cryptococcosis with a 6-week induction course of L-AMB and oral 5-FC, followed by an 8-week 800 mg oral fluconazole consolidation with a plan for a further 38 weeks of 400 mg daily to complete a total 12 month course. In addition to antifungal therapy, azithromycin, high-dose trimethoprim-sulfamethoxazole, and valganciclovir were administered for *Mycobacterium avium* complex (MAC) prophylaxis, empiric PJP treatment, and CMV viraemia, respectively. The latter two agents were later ceased due to a progressive pancytopenia. The patient was changed to atovaquone for PJP treatment and valganciclovir was recommenced later in his clinical course in response to a rising CMV viral load, again without a clear end-organ focus. Bictegravir/emtricitabine/tenofovir alafenamide was commenced after anti-fungal induction therapy was completed to reduce the risk of Immune Reconstitution Inflammatory Syndrome (IRIS); an undetectable HIV viral load was achieved by day 25 of anti-retroviral treatment. With regard to HLH, the patient's ferritin count was noted to improve from 13,300 *μ*g/L to 575 *μ*g/L following intravenous immunoglobulin, cryptococcal treatment, and management of his secondary infections. This improvement occurred prior to the commencement of antiretroviral therapy.

The patient was discharged home with no focal neurology.

## 3. Discussion

*C. neoformans* is an environmental basidiomycete fungus that typically causes opportunistic disease in immunosuppressed patients and is most frequently detected in blood, CSF, and respiratory specimens. Cryptococcal species are much less frequently encountered in urinary culture than *Candida* species [3, 7]. In one case study, a cryptococcoma was identified in a renal transplant patient with persistently culture-negative urine specimens; direct biopsy and culture of the lesion subsequently grew *C. neoformans* [2]. Cryptococcal prostatitis has been recently described in an HIV-infected individual, culture-positive for *C. neoformans* in both pulmonary and prostatic sites but culture-negative in blood and CSF specimens [8]. It appears to be exceptionally rare for cryptococcuria to herald disseminated cryptococcal infection in HIV-negative individuals, as was the case in our first patient [9–11].

Cryptococcal bone marrow infiltration is also an infrequently reported phenomenon [12, 13]. A seminal paper described the antemortem diagnosis of disseminated cryptococcosis made on a bone marrow specimen, proving that such infiltration was possible [14]. A retrospective series from 1992 to 1996 reviewed 1225 bone marrow specimens from HIV patients, and of 31 specimens positive for mycobacteria and fungi, only one marrow grew *C. neoformans* [15]. Sparse case reports have identified cryptococcal bone marrow infiltration in a young immunocompetent male [16], a patient with a new HIV diagnosis [17], and a patient with previously diagnosed HIV 8 years prior who presented with a CD4 lymphocyte count of 26 cells/*μ*L [18]. Despite the relative frequency of concomitant cryptococcal and HIV infections, cryptococcal bone marrow infiltration remains rarely reported in the literature.

HLH associated with cryptococcosis is a rare entity. The phenomenon has previously been described in the setting of pulmonary and CNS infections and, in this context, is an acquired immune dysregulation response to infection and inflammation [19, 20]. The first reported case of HLH associated with cryptococcal infection was in a case report from 1998 detailing a child with cryptococcal meningoencephalitis whose clinical course was complicated by persistent fever, cytopenia, hypercytokinaemia, liver function test (LFT) derangement, and a bone marrow biopsy suggestive of the haemophagocytic syndrome. In this instance, the child unfortunately died of acute respiratory failure attributed to HLH. The second known case was a 59-year-old male diagnosed with pulmonary cryptococcosis with a past medical history significant for type 2 diabetes mellitus and current smoking who also developed persistent fevers, cytopenia, LFT derangement, and a bone marrow examination consistent with HLH. In this instance, the patient was managed with corticosteroids, in addition to anti-fungal therapy for his cryptococcal infection, and his HLH resolved.

HLH also has known associations with HIV infection. A recent review of case reports from January 2005 to April 2021 identifying 81 adults with both HIV and HLH found the majority of patients had a secondary infectious agent thought to have triggered HLH. The most common of these triggering infections were viral, including human herpesvirus 8 and Epstein–Barr virus; though, notably, fungal infections including *Histoplasma capsulatum*, *Pneumocystis jirovecii*, and one case of *Cryptococcus neoformans* meningitis were also featured [21]. In our second patient, we propose that resolution of clinical and laboratory parameters of HLH appeared to coincide with effective anti-cryptococcal treatment, prior to commencement of antiretrovirals or the reduction in HIV viraemia, suggesting the HLH was not driven by uncontrolled HIV but potentially by the cryptococcal infection.

The *Cryptococcus neoformans* complex occurs worldwide when compared to *Cryptococcus gattii*, which has a more restricted geographical location and is often associated with immunocompetent states [22]. In Australia, the *Consensus guidelines for the diagnosis and management of cryptococcosis and rare yeast infections in the haematology/oncology setting 2021* recommend that treatment agent and duration should be based on the location of infection, focusing on the most common presentations of CNS and pulmonary cryptococcosis [23]. These guidelines follow the assumption that treatment centres have access to a full complement of antifungal agents and adequate clinical staff and laboratory facilities, which may not be as easily accessible elsewhere. The combination of intravenous L-AMB and 5-FC comprises typical induction therapy over 2 to 6 weeks for CNS and severe pulmonary and concurrent CNS/pulmonary infections. Whilst these guidelines are primarily targeted at oncology/haematology cohorts, the authors acknowledge that the recommendations may be extrapolated to other populations. They also acknowledge the lack of available research to guide non-CNS, non-pulmonary cryptococcosis. The European AIDS Clinical Society (EASC) guidelines for *C. neoformans* largely mirror the Australian consensus guidelines but suggest a two-week induction phase followed by eight weeks of consolidation therapy then secondary prophylaxis for 12 months [24].

Both of our cases demonstrate that cryptococcal infection can have unusual presentations. Our first case exemplified this, as the patient's *C. neoformans* infection manifested following a urological procedure, with an absence of pulmonary/CNS disease, and was initially treated with empirical therapy targeted at *Candida* species. Although the patient was not heavily immunosuppressed, his interferon therapy and fibrotic myelofibrosis may have contributed to this atypical presentation. There is currently a paucity of evidence to guide the management of non-pulmonary, non-CNS infections and especially limited guidance for the management in non-HIV, non-solid organ transplant (SOT) recipients. Duration of therapy for non-HIV, non-SOT patients is largely extrapolated from studies of HIV and SOT patients, and clinicians must utilise their own judgement when developing management plans for these patients. This cohort of non-HIV, non-SOT patients tends to have worse outcomes following cryptococcal infection, thought largely to be secondary to a lower index of suspicion for infection given atypical clinical presentations, thus resulting in delayed diagnosis [23].

Our second case, the young male with HIV-associated disseminated cryptococcosis, presented in a more typical fashion and was managed with an extended induction phase of dual antifungal therapy given his extensive disease. The Australian guidelines acknowledge the importance of individualised care in unique cryptococcal presentations given the paucity of evidence outside of the haematological/oncological setting [23]. These cases highlight the importance of clinicians considering the genitourinary tract and bone marrow as possible sites of cryptococcal infection when selecting appropriate diagnostic investigations and subsequent management.

## Figures and Tables

**Table 1:** Summary of investigations.

	Microscopy	Fungal culture	Cryptococcal lateral flow antigen assay	Cryptococcal nucleic acid amplification test
Blood	Case 1: budding yeast seen Case 2: budding yeast seen	Case 1: culture positive for *C. neoformans* complex Case 2: culture positive for *C. neoformans* complex	Case 1: detected, peak titre 1 : 16 Case 2: detected, peak titre >1 : 1024	Case 1: not performed Case 2: not performed

Urine	Case 1: budding yeast seen Case 2: negative	Case 1: culture positive for *C. neoformans* complex Case 2: culture negative	Case 1: not performed Case 2: not performed	Case 1: not performed Case 2: not performed

CSF	Case 1: negative Case 2: India ink stain positive, moderate budding yeast seen	Case 1: culture negative Case 2: positive for *C. neoformans* complex	Case 1: negative Case 2: positive, peak titre >1 : 1024	Case 1: not detected Case 2: detected

Bone marrow	Case 1: negative Case 2: positive (Grocott stain)	Case 1: culture negative Case 2: culture positive for *C. neoformans* complex	Case 1: not performed Case 2: not performed	Case 1: not performed Case 2: not performed

## References

[1] Rajasingham R. (2022). Cryptococcosis, BMJ Best Practice. https://bestpracticebmj-com.ap1.proxy.openathens.net/topics/en-gb/917/pdf/917/Cryptococcosis.pdf.

[2] Muranda A. Z., Greeff L., Sathekge M. M., Lengano T., Karusseit V. O. L. (2018). Cryptococcoma of a transplanted kidney in a patient presenting with recurrent urinary tract infection: a case report. *BMC Nephrology*.

[3] Dash M., Chayani N., Sarangi G. (2014). An overview of invasive fungal infections in immuno-compromised hosts. *International Journal of Infectious and Tropical Diseases*.

[4] Pappas P. G. (2013). Cryptococcal infections in non-HIV-infected patients. *Transactions of the American Clinical and Climatological Association*.

[5] Telles J. P., de Andrade Perez M., Marcusso R., Correa K., Teixeira R. F. A., Tobias W. M. (2019). Hemophagocytic syndrome in patients living with HIV: a retrospective study. *Annals of Hematology*.

[6] Fardet L., Galicier L., Lambotte O. (2014). Development and validation of the HScore, a score for the diagnosis of reactive hemophagocytic syndrome. *Arthritis and Rheumatology*.

[7] Salazar-Leal J. I., Ramírez-Montelongo S. M., López Luis B. A. (2019). Clinical significance of nosocomial Cryptococcus laurentii in urine: a case series. *Infection Control and Hospital Epidemiology*.

[8] Xu L., Tao R., Zhao Q., Cheng J., Zhu B. (2019). An AIDS patient with urine retention. *BMC Infectious Diseases*.

[9] Kiertiburanakul S., Sungkanuparph S., Buabut B., Pracharktam R. (2004). Cryptococcuria as a manifestation of disseminated cryptococcosis and isolated urinary tract infection. *Japanese Journal of Infectious Diseases*.

[10] Severo C. B., Pinto G. L., Sotilli J. (2011). Cryptococcuria as manifestation of disseminated cryptococcosis: staib agar as a selective identification medium. *Mycoses*.

[11] Weiss Z. F., DiCarlo J. E., Basta D. W. (2022). Hidden in plain sight: urinary Cryptococcus neoformans missed by routine diagnostics in a patient with acute leukemia. *Annals of Clinical Microbiology and Antimicrobials*.

[12] Ashwini B. R., Raghupathi A. R., Srinarthan A. (2014). Cryptococcososis of bone marrow: a case report with review of literature. *Journal of Clinical and Diagnostic Research*.

[13] Charles M. G. K., Carlsen E. D. (2022). Marrow cryptococcosis in an autologous stem cell transplant patient after standard therapy for cryptococcal meningitis. *J Hematopathol*.

[14] Robert F., Durant J. R., Gams R. A. (1977). Demonstration of Cryptococcus neoformans in a stained bone marrow specimen. *Archives of Internal Medicine*.

[15] Talbot E. A., Reller L. B., Frothingham R. (1999). Bone marrow cultures for the diagnosis of mycobacterial and fungal infections in patients infected with the human immunodeficiency virus. *International Journal of Tuberculosis and Lung Disease*.

[16] Donald S., Kakkar N., Loomba V. (2020). Disseminated cryptococcosis in a young immunocompetent male. *Indian J Hematol Blood Transfus*.

[17] Dharwadkar A., Vimal S., Buch A. C., Panicker N. K. (2014). HIV infection presenting as bone marrow cryptococcosis. *Advanced Biomedical Research*.

[18] Vechi H. T., Theodoro R. C., de Oliveira A. L. (2019). Invasive fungal infection by Cryptococcus neoformans var. grubii with bone marrow and meningeal involvement in a HIV-infected patient: a case report. *BMC Infectious Diseases*.

[19] Singh P. K., Kodati R., Rohilla M., Sharma P. (2019). Hemophagocytic lymphohistiocytosis: a rare association with pulmonary cryptococcosis. *BMJ Case Reports*.

[20] Numata K., Tsutsumi H., Wakai S., Tachi N., Chiba S. (1998). A child case of haemophagocytic syndrome associated with cryptococcal meningoencephalitis. *Journal of Infection*.

[21] Tabaja H., Kanj A., El Zein S., Comba I. Y., Chehab O., Mahmood M. (2022). A review of hemophagocytic lymphohistiocytosis in patients with HIV. *Open Forum Infectious Diseases*.

[22] Kwon-Chung K. J., Fraser J. A., Doering T. L. (2014). Cryptococcus neoformans and Cryptococcus gattii, the etiologic agents of cryptococcosis. *Cold Spring Harbor Perspectives in Medicine*.

[23] Chang C. C., Hall V., Cooper C. (2021). Consensus guidelines for the diagnosis and management of cryptococcosis and rare yeast infections in the haematology/oncology setting, 2021. *Internal Medicine Journal*.

[24] European Aids Clinical Society (2021). European AIDS clinical society guideline version 11.0. https://www.eacsociety.org/media/final2021eacsguidelinesv11.0_oct2021.pdf.

